# Changing Trends in Gastric Cancer Surgery

**DOI:** 10.4274/balkanmedj.2015.1461

**Published:** 2017-01-05

**Authors:** İlter Özer, Erdal Birol Bostancı, Murat Ulaş, Yusuf Özoğul, Musa Akoğlu

**Affiliations:** 1 Department of Gastroenterological Surgery, Türkiye Yüksek İhtisas Training and Research Hospital, Ankara, Turkey

**Keywords:** Gastric cancer, lymph node dissection, bursectomy, splenectomy, minimally invasive surgery

## Abstract

Gastric cancer is one of the most common causes of cancer-related death. It requires multimodal treatment and surgery is the most effective treatment modality. Radical surgery includes total or subtotal gastrectomy with lymph node dissection. The extent of lymphadenectomy still remains controversial. Eastern surgeons have performed D2 or more extended lymphadenectomy while their Western colleagues have performed more limited lymph node dissection. However, the trend has been changing in favour of D2 lymph node dissection in both hemispheres. Currently, D2 is the recommended type of lymphadenectomy in experienced centres in the west. In Japan, D2 lymph node dissection is the standard surgical approach. More extensive lymphadenectomy than D2 has not been found to be associated with improved survival and generally is not performed. Bursectomy and splenectomy are additional controversial issues in surgical performance, and trends regarding them will be discussed. The performance of bursectomy is controversial and there is no clear evidence of its clinical benefit. However, a trend toward better survival in patients with serosal invasion has been reported. Routine splenectomy as a part of lymph node dissection has largely been abandoned, although splenectomy is recommended in selected cases. Minimally invasive surgery has gained wide popularity and indications for minimally invasive procedures have been expanding due to increasing experience and improving technology. Neoadjuvant therapy has been shown to have beneficial effects and seems necessary to provide a survival benefit. Diagnostic laparoscopy should be kept in mind prior to treatment.

Gastric cancer is a common cause of cancer-related death worldwide and requires multidisciplinary management. Surgery is the main treatment modality in gastric cancer treatment. Improvements in surgical technique, patient care and perioperative therapies have resulted in continuous changes in the surgical approach. The most controversial topic in surgery for gastric cancer is the extent of lymph node dissection (LND). While surgeons in Japan perform extended lymphadenectomy, surgeons in the West commonly prefer a more limited LND. This review is focused on controversial topics and trends in surgical performance. Bursectomy and splenectomy, as a part of lymphadenectomy, remain controversial and have been performed without clear evidence of survival benefit. This review discusses trends in lymphadenectomy, bursectomy and splenectomy, and summarizes the trend toward minimally invasive surgery (MIS). In addition, endoscopic techniques, diagnostic laparoscopy and neoadjuvant therapy are briefly mentioned.

## LYMPHADENECTOMY

Gastric cancer is a worldwide health problem and the main treatment modality is radical surgery including gastrectomy with lymphadenectomy. However, the extent of LND is still controversial despite numerous studies. The rationale behind lymphadenectomy is based on the lymphatic spread of cancer. Gastric cancer frequently metastasizes to regional lymph nodes, even in the early stages of the disease. The risk of lymph node metastasis gradually increases with increasing depth of penetration. For adenocancers invading the submucosa, the rate of lymph node metastasis may reach 20%, and for T2 tumours the metastasis rate may exceed 50% (1). Trends in the extent of LND seem to be moving in opposite directions; in other words, practices in the east and west have been approaching each other.

Gastric cancer is a common health problem that may result in a patient’s death. In Japan, Japanese surgeons have made great efforts to achieve a better survival rate. The standard operation technique was defined by the Japanese Research Society for Gastric Cancer in 1962 (2). Radical surgery is performed in relation to five major factors: stomach wall penetration, lymph node metastasis, peritoneal metastasis, invasion to adjacent structures and liver metastasis (2). Japanese surgeons preferred systematic LND and lymphadenectomy techniques, based on studies of lymphatic flow, risk of metastasis and survival benefit (2). Regional lymph nodes were categorized into four groups: N1, N2, N3 and N4. The extent of lymphadenectomy was expressed using the letter R. The standard LND was R2, which included the removal of all the lymph nodes in groups N1 and N2. The N categories depended on the location of the tumour. Middle colic artery (No: 15) and para-aortic (No: 16) lymph nodes were classified as N4 for all locations. R2 (currently D2) lymphadenectomy is the standard LND technique used by Japanese surgeons since the 1960s. Lypmhadenectomies beyond R2 have been performed in Japan as well. In 1989, the Maruyama computer (3) program was created to calculate the risk of metastasis in each lymph node station using eight variables (age, gender, Borrmann classification, depth of invasion, size, location, position and histological classification). The program was created based on data from 3843 patients and it was also possible to estimate the five-year survival rate according to the location and lymph node station involved. The rationale for extensive lymphadenectmy in surgery for gastric cancer is based on several findings in clinical studies. These include an association between survival and the number of metastatic lymph nodes (4), an incidence of metastases para-aortic lymph node involvement of over 20% in some studies (5) and higher survival rates in some patients with para-aortic node metastasis who had undergone dissection of those nodes (6).

In 1998, the second English edition of the Japanese Classification of Gastric Carcinoma was published (7). According to this classification, regional lymph node stations (Table 1) were classified into three levels (or compartments) depending on the location of the tumour. The stations in the compartments could change according to the location of the primary tumour. For example, No: 2 lymph nodes were classified as N1 for tumours in the upper third, but as distant metastasis for those in the lower third of the stomach. The grade of lymph node metastasis (N1-3) was expressed according to the level (compartment) of the lymph node station involved. D2 LND was defined as the removal of all N1 and N2 nodes, and D3 LND was defined as dissection of the N3 nodes in addition to N1 and N2 lymph nodes.

Once the gastric tumour invades the subserosa, the serosa or the adjacent structures, para-aortic lymph nodes can be involved (7,8). Because the five-year survival of patients with para-aortic lymph node metastases can reach 20% after extended LND (6), extended LND had been performed by Japanese surgeons since the 1980s for tumours invading the subserosa, serosa and adjacent structures (8). However, a large-scale prospective study concerning the long-term benefit of para-aortic LND had not been performed until the study by Sasako et al. (8) in 2008. The authors reported the final results of the randomized trial conducted by the Japan Clinical Oncology Group (JCOG9501). The interim analysis of this trial showed similar short-term results (9). The five-year survival rates after D2 LND alone and D2 lymphadenectomy plus para-aortic LND were 69.2% and 70.3%, respectively. The five-year recurrence-free survival rates were 62.6% and 61.7%, respectively. No improvement was observed in overall or recurrence-free survival after D2 lymphadenectomy plus para-aortic LND, and recurrence rates in the lymph nodes were similar. Patients with para-aortic nodal metastasis showed a five-year survival rate of 18.2%. The authors concluded that extended D2 lymphadenectomy plus para-aortic LND should not be performed for gastric cancer patients with T2b, T3 or T4 disease.

A prospective observational study was performed in our clinic to evaluate the outcomes in gastric cancer patients undergoing D2 or D3 lymphadenectomy (10). The five-year survival rates after D2 and D3 LND were 42.6% and 38.6%, respectively. Moreover, with respect to tumour stage, survival rates did not differ according to whether patients had undergone D2 or D3 dissection. The three-year survival rate for patients with para-aortic metastases was 18.8%.

Eastern surgeons still consider D2 dissection the standard operation and refuse to conduct studies comparing D1 and D2 dissection, as they regard these studies as unethical. However, in the west, no large randomized controlled trials addressed the extent of LND in gastric cancer patients until the late 1990s. The widely applied LND procedures rarely exceeded D1. In the late 1980s, Dent et al. (11) conducted a randomized comparison of R1 and R2 lymphadenectomies that included 43 patients; the study found that R2 surgery was associated with high morbidity and suggested that this procedure should not be performed as the survival advantage was not proven. However, during the 1990s, D2 dissection was performed by an increasing number of centres in the west and improved outcomes after D2 dissection were reported (12,13). The most important and largest-scale studies in the Western world were published in 1995 and 1996. The Dutch Gastric Cancer Trial (14) and the Medical Research Council (MRC) Trial (15) published early results after D1 and D2 LND. Both trials found significantly higher morbidity and mortality rates after D2 dissection. However, especially in the MRC trial, the higher morbidity was largely attributed to the pancreatic resections and splenectomies that were done as a part of D2 dissection for middle and upper tumours. In 1999, the long-term results of these two prospective randomized trials were published (16,17) and no long-term survival advantage was found after D2 LND. There was no improvement in long-term results with regard to overall and disease-free survival or recurrence risk.

In the Dutch trial, splenectomy was found to be associated with increased post-operative complications and reduced survival. The cumulative risk of relapse was reduced in the D2 arm, although not significantly. There was a marginally significant difference in stage II and IIIA [Union for International Cancer Control (UICC)] patients, which was attributed to stage migration by the authors. In the MRC trial, the best long-term survival was observed after D2 LND without pancreaticosplenectomy. According to the short- and long-term results of these large-scale trials, the routine performance of D2 LND was not recommended. However, these Western studies were criticized due to insufficient experience in D2 LND, a low volume of individual centres, the standardization of surgical techniques and high mortality rates after D2 LND.

In 2010 and 2011, 15-year follow-up results of the Dutch trial (18) and Japanese Gastric Cancer Association Japanese gastric cancer treatment guidelines 2010 (ver. 3) (19) were published. The Japanese and the Western approaches were becoming more similar. The 15-year follow-up results of the Dutch trial showed that D2 lymphadenectomy was associated with lower locoregional recurrence (12% vs. 22%) and gastric cancer-related death rates (37% vs. 48%) than D1 lymphadenectomy. The 15-year survival rates were 29% and 21% after D2 and D1 LND, respectively. Among patients without pancreaticosplenectomy, the survival rate after D2 LND was significantly higher than that after D1 LND (35% vs. 22%). The authors concluded that since a spleen-preserving D2 LND could be done safely, D2 is the recommended extent of lymphadenectomy for resectable gastric cancer.

In 2011, major revised points in the new Japanese classification (20) and gastric cancer treatment guidelines (19) were summarized (21). In the new classification, tumor-node-metastasis (TNM) (including organ metastasis, peritoneal metastasis and peritoneal cytology) categories were identical to those in the UICC/TNM 7th edition. Expression of the grade of nodal metastasis according to anatomic compartments was abandoned. The grade of lymph node metastasis was expressed in terms of the number of metastatic lymph nodes for the first time in Japanese classification systems. This modification of the N classification was regarded as the largest change in the history of Japanese classifications. In addition, the extent of LND definition was revised and remarkably simplified. D3 LND was not defined in the new classification due to the lack of additional survival benefit. Lymphadenectomy levels (D1, D1+ and D2) were defined for total and distal gastrectomy regardless of the location of the primary tumour (Table 2). The indications for the extent of lymphadenectomy were also defined (Table 3).

The 2015 National Comprehensive Cancer Network (NCCN) guidelines group lymph node stations differently. Perigastric nodes (Nos. 1-6) are grouped as N1, while lymph nodes 7-11 are grouped as N2. Nodal involvement beyond N2 is regarded as distant metastasis. However, in the 2010 Japanese classification, No: 7 lymph nodes (left gastric artery) were included in the N1 group, while anterior hepatoduodenal nodes (No: 12a) were included in the N2 group. Thus, the definition of the extent of LND is different. In addition, in the 2015 NCCN guidelines, the definition of D2 LND is not clear. For example, during a subtotal gastrectomy, what should surgeons do with left paracardial and splenic hilar nodes and nodes along the distal splenic artery (Nos. 2, 10 and 11d)? In addition, the 2015 NCCN guidelines state that D2 LND is considered a recommended, but not a required procedure. In the guidelines, D1 or modified D2 LND with the aim of harvesting at least 15 lymph nodes is recommended. In addition, the guidelines suggest that D2 LND should be performed by experienced surgeons in high-volume centres. We think that these recommendations pose some questions. For example, what does “recommended but not required” mean? As radical surgery provides the greatest chance for cure, can we omit a recommended operation in oncologic surgery, particularly in gastric cancer surgery? And does an individual gastric cancer patient deserve at least the supervision of an experienced surgeon?

In conclusion, the approaches for lymphadenectomies in the western and eastern world have become more similar and D2 dissection is the widely accepted level of LND for gastric cancer surgery. However, there is still a difference between practices in the two regions, as demonstrated by the use of the word “recommended” as opposed to “standard”. The new controversy may be about the extent of D2 LND.

## BURSECTOMY

Bursectomy refers to a surgical procedure that includes removal of the anterior leaf of the transverse mesocolon and the peritoneal lining of the pancreas via omentectomy. During gastric cancer surgery, bursectomy is performed to remove free cancer cells and microscopic tumour deposits in the lesser sac and greater omentum, for complete resection of disease from the pancreas, complete dissection of the subpyloric lymph nodes and a regular coeliac-based lymphadenectomy (22,23). However, there is no clear evidence of its clinical benefit and the performance of bursectomy is controversial. The Japanese Gastric Cancer Treatment Guidelines recommend bursectomy for tumours involving the serosa of the posterior gastric wall without clear evidence, while suggesting the avoidance of bursectomy for tumours without serosal invasion (19). Bursectomy may sometimes be a time-consuming and difficult procedure and may lead to procedure-related morbidity. The most important cause of morbidity is a leak due to pancreatic injury (pancreatic fistula). In addition, skeletonization of the mesocolon and pancreas may lead to adhesion formation, which may cause intestinal obstruction, afferent loop syndrome and delayed gastric emptying (22). Blouhos et al. (22) observed five intraoperative complications (spleen injury in two patients, common bile duct injury in one patient and portal vein injury in two patients) in their prospective cohort study that included 72 patients. The authors reported a post-operative complication rate of 19.4% and they observed three pancreatic fistulas (two grade As, one grade B) (22). There are various studies concerning the safety of bursectomy in the literature. In general, morbidity and mortality rates have been reported to be similar between bursectomy and non-bursectomy groups (24,25,26). However, specific complications directly related to bursectomy such as pancreatic fistula have been observed. Herbella et al. (27) stated that subclinical pancreatic fistulas could occur in up to 10% of patients after pancreatic capsule removal. Imamura et al. (24) observed greater blood loss and longer operative time in their multi-institutional randomized controlled trial. The overall morbidity was the same. Moreover, they did not observe an increased rate of pancreatic fistula in the bursectomy group. The authors concluded that bursectomy could be performed safely by experienced surgeons. The keyword in this sentence is “experienced”, as bursectomy technique can be very complicated and time-consuming with potential complications including intraoperative blood vessel injury, haemorrhage and pancreatic fistula. Special training and experience are necessary to perform a complicated surgical technique in oncologic procedures. We think that bursectomy can be performed safely in experienced centres during D2 LND.

Safety and oncologic benefit are the two primary factors required for a surgical procedure to become a standard treatment. Data regarding the long-term results are lacking and there is little evidence that bursectomy has a long-term survival benefit. There are few studies concerning the survival benefit of bursectomy. One of them was a randomized controlled trial conducted by Fujita et al. (25), the short-term results of which were mentioned above. The authors reported their interim results and stated that survival was better in the bursectomy group, although the difference was not statistically significant. Recurrence-free survival was similar among serosa-negative patients; however, bursectomy provided better survival in serosa-positive patients. The authors suggested that better survival after bursectomy was attributable to the removal of free cancer cells rather than more accurate lymphadenectomy. In contrast, Kochi et al. (26) reported that there was no improvement in overall survival after D2 gastrectomy and bursectomy in their retrospective study that included 254 patients. They reported similar post-operative complication rates, and similar overall and disease-free survival rates in the two groups. In patients with metastatic lymph nodes, the overall and disease-free survival seemed better in the bursectomy group (71.1% vs. 58.8%, and 67.6% vs. 56.6%, respectively); however, the differences were not significant. The bursectomy group had better overall and disease-free survival (67.6% vs. 61.5%, and 58.4% vs. 40.5%, respectively) in patients with serosal involvement. The differences were not significant, although the difference in disease-free survival seemed remarkable. The authors concluded that they could not recommend curative D2 gastrectomy plus bursectomy. Eom et al. (28) found that overall survival was not improved after bursectomy. Even in the subgroup of the patients with tumours with serosal involvement of the posterior wall, no survival advantage of bursectomy was observed by the authors. However, the number of patients in this subgroup was not sufficient to provide statistically relevant results.

The anatomic features of the bursa omentalis are also an important factor regarding the validity of bursectomy. The bursa omentalis is open to the abdominal cavity through the foramen of Winslow. As it is not a closed cavity, free cancer cells could potentially migrate into the abdominal cavity. Yamamura et al. (29) measured CEA and CK20 messenger RNA (mRNA) levels in the bursa omentalis and other abdominal cavities using the reverse transcription-polymerase chain reaction technique. Of the 136 patients included in the study, free cancer cells were detected in at least one sample obtained from three different cavities in 43 patients. mRNAs were detected in only 14 samples obtained from the bursa omentalis. However, in 12 of these 14 patients, mRNAs were also detected in at least one sample obtained from the other two abdominal cavities. mRNAs were detected only in the bursa omentalis in only two patients.

A meta-analysis evaluating the survival benefit of bursectomy was published in 2014 (30). To the best of our knowledge, there are few studies with a large number of patients that have evaluated the effects of bursectomy on survival. Thus, this meta-analysis included only five studies, three of which were non-randomized trials. In this meta-analysis, bursectomy was unlikely to be associated with improved overall and disease-free survival. However, in patients with serosal involvement, overall and recurrence-free survival was better in the bursectomy group, although the differences did not reach statistical significance.

The most recent study was published in 2015 in which Hirao et al. (31) reported their long-term results after prophylactic bursectomy. The five-year OS was 77.5% for the bursectomy group and 71.3% for the non-bursectomy group. The bursectomy procedure was found to be an independent prognostic factor for good survival according to Cox multivariate analysis. Bursectomy provided significantly better overall survival for tumours in the middle or lower third, compared to the non-bursectomy group. The five-year survival was 80.7% for the bursectomy group and 70.7% for the non-bursectomy group. Among pT3 or pT4 patients, five-year survival rates were 55.5% and 34.8%, respectively. A trend toward improved survival was observed after bursectomy for middle and lower tumours and for pathologically serosa-positive tumours. The authors concluded that bursectomy should not be regarded as a futile procedure and should not be abandoned.

Current trends seem to be moving towards not performing bursectomy. This trend cannot be considered without other trends in gastric cancer surgery. As a result of recent developments in MIS, laparoscopic gastrectomy has been increasingly performed for gastric cancer. During laparoscopy, the performance of bursectomy is a significant technical challenge, and this may force some surgeons to be sceptical about the clinical benefits of bursectomy. However, in most of the studies mentioned above there was a trend of better survival rates in patients with serosal invasion. Bursectomy was previously recommended for tumours with serosal invasion. The new Japanese Gastric Cancer Treatment Guidelines published in 2011 limit this recommendation to cases with serosal penetration of the posterior gastric wall (19).

A multicentre randomized controlled trial involving 1.000 patients is currently being carried out by the Japan Clinical Oncology Group to compare the long-term results of gastrectomy plus D2 LND with and without bursectomy among cT3 and cT4 patients.

## SPLENECTOMY

Distal pancreatectomy as part of LND has been abandoned because of its negative effects on short- and long-term results. However, routine splenectomy remains controversial. The Japanese Gastric Cancer Association (7) recommended simultaneous splenectomy during total gastrectomy to facilitate removal of splenic hilar lymph nodes in advanced gastric cancer involving the upper third of the stomach. However, some reports have shown no survival benefit from routine splenectomy (32,33). Kunisaki et al. (33) suggested that dissection of the splenic hilar lymph nodes had little survival benefit, probably due to simultaneous metastatic involvement of lymph nodes in far areas, such as para-aortic lymph nodes. They recommended splenectomy only for tumours with splenic invasion and in the presence of metastatic lymph nodes extending to the spleen. Meta-analysis conducted by Yang et al. (34) revealed that splenectomy did not provide a long-term survival advantage when compared to preservation of the spleen. In addition, Zhu et al. (35) reported that the presence of splenic hilar nodal metastasis was associated with poorer prognosis when compared with the survival of patients with negative nodes in the splenic hilum. They found that in patients with metastatic splenic hilar nodes, survival after R0 resection was similar to that after R1-2 resection. Metastasis in splenic hilar lymph nodes was found to be an independent predictive factor for poor survival. The authors concluded that it should be considered an incurable factor. Since these studies showing the ineffectiveness of splenectomy, routine splenectomy has largely been abandoned. Kosuga et al. (36) found that the risk of splenic hilar lymph node metastasis tended to be higher in patients with Borrmann type 4 cancers and with tumours on the greater curvature. They suggested that splenectomy might have a long-term survival advantage for patients with such tumours. The Japanese Gastric Cancer Treatment Guidelines (ver. 3) (19) recommend splenectomy for tumours localized along the greater curvature and in the presence of metastasis in 4sb lymph nodes.

## MINIMALLY INVASIVE SURGERY

### Laparoscopic surgery

Laparoscopic surgery has been increasingly used for the treatment of gastric cancer in the last two decades. In 1994, Kitano et al. (37) performed the first laparoscopy-assisted distal gastrectomy with LND. Since then, laparoscopic procedures have gained widespread popularity and were pioneered especially in Korea, Japan and China. Short-term results after laparoscopic surgery are well known and widely accepted; however, in oncologic procedures, equivalency in long-term oncologic results should be provided. Many studies evaluating the safety and efficacy of these procedures have shown encouraging results and more complicated and technically challenging operations have been performed. Distal gastrectomy, total gastrectomy, pylorus-preserving gastrectomy and proximal gastrectomy with an appropriate lymphadenectomy can be performed laparoscopically with good post-operative results. However, there are controversies about some issues including an adequate lymphadenectomy and the long-term oncologic results. Oncologic principles, including minimal manipulation of the tumour, achieving negative margins and performing an adequate LND, are mandatory.

Laparoscopic distal gastrectomy (LDG) has rapidly gained wide popularity for early gastric cancer, as several reports have shown favourable short- and long-term results (38). However, laparoscopic total gastrectomy (LTG) is regarded as a challenging procedure because of the difficulty of reconstruction and performing a complete LND.

The generally accepted indication of MIS is early gastric cancer without clinical evidence of metastatic lymph nodes. Several randomized controlled trials evaluating laparoscopic gastrectomy (LG) have shown favourable results (39,40). The indications for this procedure have been expanding due to increasing experience and improving technology.

There are several studies and meta-analyses concerning the short- and long-term results of laparoscopic gastrectomies. In 2012, Vinuela et al. (41) reported the results of their meta-analysis, which included high-quality non-randomized studies and randomized controlled trials comparing LDG and open distal gastrectomies. Operative time was longer in the LDG group. Overall complications, medical complications, minor surgical complications, estimated blood loss and hospitalization were lower in the LDG group. Major complications and mortality were similar. Although the proportion of patients with fewer than 15 harvested lymph nodes was similar, the number of harvested nodes was significantly lower in the LDG group. However, the ODG group had a higher proportion of D2 dissections. The authors suggested that LDG was safe and favourable with regard to short-term results. The interim results of the Korean Laparoscopic Gastrointestinal Surgery Study (KLASS) trial were published in 2010 (42). The purpose of the study was to evaluate the short- and long-term results of laparoscopy-assisted distal gastrectomy (LADG) in the treatment of early gastric cancer. In the interim report, they found similar morbidity and mortality rates. Post-operative complications were seen in 10.5% (17/179) and 14.7% (24/163) of the patients in the LADG and ODG groups, and the post-operative mortality rates were 1.1% (2/179) and 0% (0/163), respectively. The authors concluded that the trial was acceptable with regard to short-term outcomes. The long-term results of this trial are awaited.

Studies reporting acceptable long-term results after LDG have also been performed (38,43). In the prospective randomized study by Huscher et al. (38), LDG was found to be safe and feasible in terms of short- and long-term outcomes. D2 dissection was performed in most of the patients. Pugliese et al. (43) performed MIS with D2 dissection on all patients enrolled in their study. They stated that subtotal gastrectomy and D2 LND performed by MIS was safe and the long-term results were acceptable.

LTG is a more complex surgical procedure than LDG. It is technically demanding and has not been widely established. However, this procedure has been increasingly performed and results are beginning to be reported. Two recent systematic reviews (44,45) revealed that LTG was safe and acceptable with regard to the number of harvested nodes, complications, mortality and long-term results. Moreover, blood loss was lower, the beginning of oral intake was earlier and hospital stay was shorter. Although long-term oncologic results were similar in the evaluated studies, there were no prospective randomized studies with a significant number of patients, thus a definite conclusion cannot be drawn. Several reports favour LTG with lymphadenectomy with good short- and long-term outcomes; however, these studies are not prospective randomized trials with large numbers of patients (46,47).

In conclusion, further studies with adequate numbers of patients are required to evaluate the oncologic results of LG. The results of two large-scale studies from Korea and Japan are awaited. The technically demanding aspects of this procedure, including reconstruction and lymphadenectomy, will be overcome with increasing experience.

## ROBOTIC GASTRECTOMY

The laparoscopic approach has some technical limitations including a two-dimensional view, decreased sense of touch, limited range of motion and physiologic tremor. Robotic systems overcome these limitations by providing a three-dimensional magnified view, more comfortable surgeon position, ergonomic comfort and elimination of physiologic tremor. These advantages are especially important during the challenging parts of the operation, such as LND and reconstruction. Studies have shown that robotic surgery (RS) is safe and feasible.

Coratti et al. (48) reported the results of 98 patients treated by RS. In addition, they reported the long-term results of their series. They found comparable short-term results regarding post-operative complications and mortality and suggested that RS could be performed with oncologic results similar to open and laparoscopic surgeries. Meta-analysis by Xiong et al. (49) revealed that RS was associated with less blood loss than LG. They found no significant differences regarding surgical complications, mortality, conversion rate, hospital stay or the number of harvested nodes. However, operative time was longer in the RS group. Although short-term results are favourable, long-term results of RS are required. In addition, the higher cost remains a disadvantage of RS.

With increasing experience and technological improvements in MIS, single-incision laparoscopic surgery and natural orifice transluminal endoscopic surgery are being investigated as options for treating early gastric cancer. However, studies evaluating their short- and long-term results are needed.

## ENDOSCOPIC TECHNIQUES

Endoscopic techniques, including endoscopic mucosal resection (EMR) and endoscopic submucosal dissection (ESD), can be performed for carefully selected forms of early gastric cancer. Endoscopic approaches are widely used in Japan and Korea as a result of the high proportion of early gastric cancers. However, these techniques are also increasingly used in specialized centres in the West. The Japanese Gastric Cancer Treatment Guidelines (19) recommend endoscopic resection for tumours that have a very low possibility of lymph node metastasis and are suitable for en-bloc resection. Standard criteria for EMR or ESD include a differentiated-type adenocarcinoma without ulcerative findings, T1a tumours and lesions ≤2 cm in diameter (19).

## DIAGNOSTIC LAPAROSCOPY

Despite detailed preoperative imaging, peritoneal carcinomatosis could not be identified until surgical exploration leading to a high rate of unnecessary surgery in gastric cancer patients. Diagnostic laparoscopy has been considered for identifying occult metastatic disease that is not detected preoperatively. Thus it has the potential of avoiding unnecessary laparotomy and changing the treatment modality. During laparoscopy, free intraperitoneal cancer cells in the absence of macroscopic peritoneal seeding can be detected using cytologic examination of the peritoneal lavage samples.

Positive peritoneal cytology has been demonstrated to be a strong prognostic marker of increased recurrence and poor survival in patients with gastric cancer (50). The NCCN guidelines and the 7th edition of the American Joint Committee on Cancer (AJCC) staging system have regarded positive peritoneal cytology as M1 disease. According to the systematic review by Leake et al. (51), dignostic laparoscopy was superior to conventional preoperative investigations in detecting peritoneal carcinomatosis and diagnostic laparoscopy changed the management of up to 59.6% of gastric cancer patients. The authors recommended diagnostic laparoscopy for patients with T3 and T4 gastric adenocarcinoma.

Diagnostic laparoscopy and cytologic examination are recommended in high-risk patients for positive cytology (T3, T4, N+) with locally advanced gastric cancer during staging laparoscopy prior to neoadjuvant or perioperative chemotherapy. Positive cytology is currently staged as M1 disease and the survival of these patients after neoadjuvant therapy and surgery is poor. The study by Lorenzen et al. (52) indicated that neoadjuvant chemotherapy did not greatly influence the cytology status. Elimination of free cancer cells by chemotherapy seemed to improve short-term prognosis. However, this effect was limited, with a poor long-term survival.

In conclusion, diagnostic laparoscopy is beneficial in avoiding unnecessary laparotomy and changing the treatment modality prior to surgery or initiation of neoadjuvant chemotherapy.

## NEOADJUVANT THERAPY

Multimodal treatment modalities, especially neoadjuvant treatment, have gained increasing popularity for advanced gastric cancer patients. The only potential curative treatment modality for gastric cancer is surgical resection with adequate lymphadenectomy. However, the results after surgery for advanced stages are still unsatisfactory. Thus, additional treatments such as neoadjuvant therapy seem necessary for providing a survival benefit. Neoadjuvant treatment has the potential advantages of downstaging and downsizing the primary tumour, eliminating micrometastases, increasing the possibility of an R0 resection and determining whether the tumour is sensitive to chemotherapy.

Neoadjuvant chemotherapy is frequently applied in the form of perioperative therapy. Perioperative therapy includes preoperative neoadjuvant chemotherapy prior to surgery and adjuvant chemotherapy post-operatively. Three large prospective randomized trials evaluated the effects of perioperative therapy (53,54,55). One of them is the MAGIC study by Cunningham et al. (53). Patients were randomized to either a perioperative chemotherapy and surgical resection group or surgery alone. The proportion of stage T1 and T2 tumours was greater and nodal disease was less advanced in the perioperative-chemotherapy group. Progression-free survival and overall survival were significantly higher in the perioperative-chemotherapy group than in the surgery group. Five-year survival rates were 36.3% in the perioperative-chemotherapy group and 23.0% in the surgery group. The authors concluded that perioperative chemotherapy improved overall and progression-free survival among patients with resectable adenocarcinoma of the stomach, lower oesophagus or gastroesophageal junction, as compared with surgery alone. In the other large-scale study (FNCLCC and FFCD), Ychou et al. (54) conducted a phase 3 trial to compare surgical resection with or without perioperative chemotherapy using fluorouracil and cisplatin in patients with resectable adenocarcinoma of the lower oesophagus, gastroesophageal junction or stomach. They observed a significantly increased curative resection rate, and better results with regard to disease-free and OS among patients in the perioperative chemotherapy group. However, the EORTC 40954 trial, which randomized patients with locally advanced (UICC stages 3 and 4) gastric and oesophagogastric junction adenocarcinomas to either chemotherapy followed by surgery or surgery alone, was stopped owing to poor accrual. This study showed increased R0 resection rates (81.9% vs 66.7%); however, it failed to show any survival benefit (55). A meta-analysis by Xiong et al. (56), which included 12 prospective randomized trials, was published in 2014. The meta- neoadjuvant chemotherapy was safe and feasible, and could significantly downstage the tumour and improve the R0 resection rate. A slightly improved survival rate was found in patients with advanced gastric and gastroesophageal cancer. Another meta-analysis by Ronellenfitsch et al. (57) revealed prolonged survival in patients with gastroesophageal adenocarcinoma. They also observed a larger survival advantage for tumours of the gastroesophageal junction than for other localizations.

Neoadjuvant chemoradiotherapy has also gained increasing interest after studies focusing on preoperative radiotherapy or chemoradiotherapy (58,59,60). Zhang et al. (58) conducted a prospective randomized trial on preoperative radiotherapy, which included 370 patients with adenocarcinoma of the cardia. The authors randomly assigned the patients to either preoperative radiotherapy prior to surgery or surgery alone. They observed a significantly improved curative resection rate and long-term survival in the neoadjuvant radiation group. Stahl et al. (59) compared the results of preoperative chemoradiotherapy and preoperative chemoradiotherapy in patients with locally advanced adenocarcinoma of the lower oesophagus or gastric cardia. Median survival was 21.1 months in the preoperative chemotherapy group and 33.1 months in the preoperative chemoradiotherapy group. The study was closed early without a statistical significance. However, a survival advantage for preoperative chemoradiotherapy compared with preoperative chemotherapy was observed. Finally, in the Chemo-Radiotherapy for Oesophageal cancer followed by Surgery Study trial, patients were randomized to chemoradiotherapy followed by surgery and surgery alone (60). A pathological complete response was achieved in 29% of the patients after chemoradiotherapy. The rate of R0 resection was significantly higher in the chemoradiotherapy + surgery group (92% vs 69%). Overall survival was significantly better (49.4 months vs 24.0 months) and post-operative morbidity and mortality rates were similar.

Neoadjuvant therapy seems to be a good option for advanced gastric cancer. NCCN guidelines recommend neoadjuvant therapy for patients with ≤T2 and N+ tumours. However, further studies and data are needed to show the definite effects of neoadjuvant therapy.

## CONCLUSION

The cornerstone of gastric cancer treatment is radical surgery. Although there is no doubt about the role of surgery, its extent remains controversial. While gastrectomy with D2 LND is the standard therapy in the Eastern Hemisphere, the extent of lymphadenectomy remains variable in the West. Lymphadenectomy of less than D1 might be performed in many Western centres. However, there is a trend toward D2 LND, especially in high-volume centres, and it has become the recommended extent of LND in experienced centres. The survival benefit of bursectomy has not been shown clearly. However, some studies have reported a trend toward better survival in patients with serosal invasion. It should be avoided in cases of T1 and T2 tumours. Long-term results of the trial being carried out by the Japan Clinical Oncology Group are awaited. Routine splenectomy as a part of LND has largely been abandoned, although splenectomy is recommended in selected cases.

The generally accepted indication for MIS is early gastric cancer; however, indications for minimally invasive procedures have been expanding due to increasing experience and improving technology. Long-term results from large-scale studies are required. Improvements in experience, surgical technique and technology will lead to further developments in gastric cancer surgery.

Despite radical surgery, the long-term results are not satisfactory for advanced gastric cancer. Neoadjuvant therapy has been shown to have beneficial effects and seems necessary to provide a survival benefit. Diagnostic laparoscopy should be kept in mind prior to surgery and neoadjuvant therapy in selected advanced gastric cancer patients.

## Figures and Tables

**Table 1:**
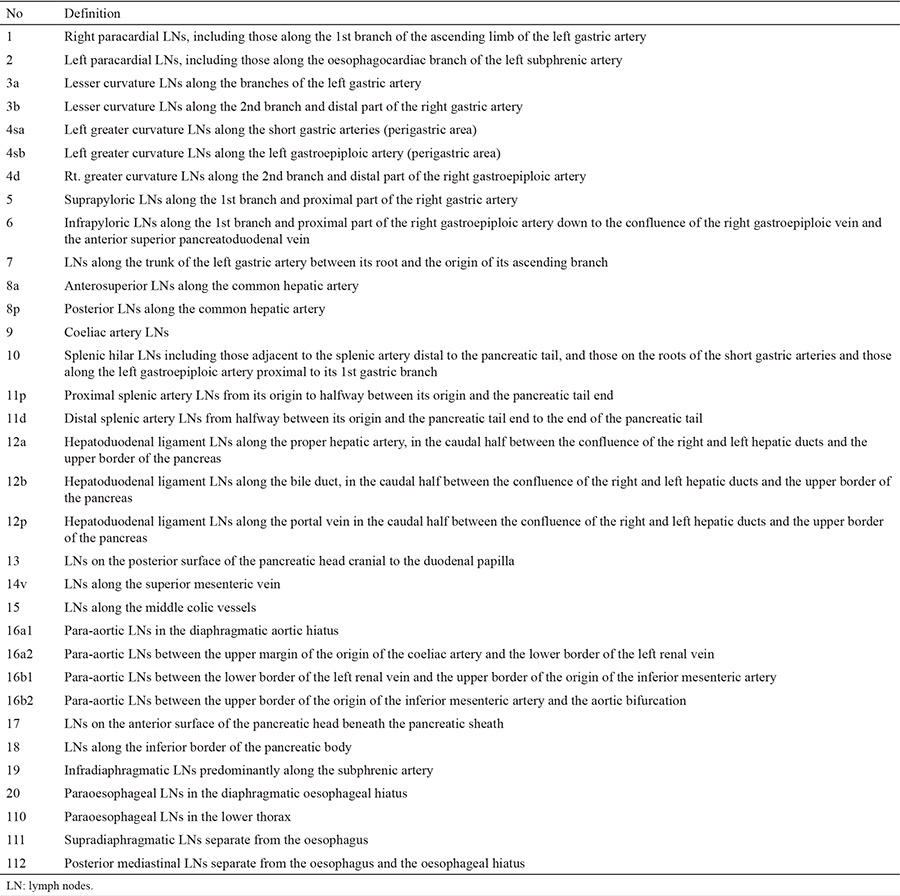
Lymph node stations (Japanese Classification of Gastric Carcinoma)

**Table 2:**
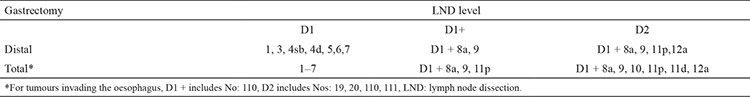
Lymph nodes to be harvested in gastric cancer surgery (Japanese Gastric Cancer Treatment Guidelines (ver. 3)

**Table 3:**
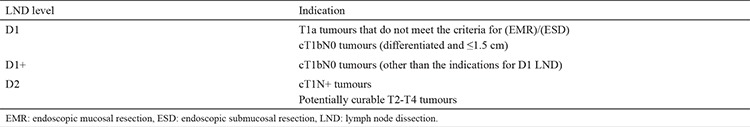
Indications for LND levels (Japanese Gastric Cancer Treatment Guidelines (ver. 3)
